# Hypoglossal nerve monitoring, a potential application of intraoperative nerve monitoring in head and neck surgery

**DOI:** 10.1186/1477-7819-11-225

**Published:** 2013-09-12

**Authors:** Carlos S Duque, Andres F Londoño, Adriana M Penagos, Diana P Urquijo, Juan P Dueñas

**Affiliations:** 1 Department of Surgery, Hospital Pablo Tóbon Uribe, Calle 78B No 69-240, Barrio Robledo, Medellin, Colombia; 2 Department of Otolaryngology, Hospital Pablo Tobón Uribe, Calle 78B No 69-240, Barrio Robledo, Medellin, Colombia; 3 Department of Otolaryngology, Facultad de Medicina, Universidad de Antioquia, Carrera 51D con Calle 62, Medellin, Colombia; 4 Clinica Las Americas, Instituto de Cancerología, Carrera 80 Diagonal 75B No 2A-80/240, Medellin, Colombia

**Keywords:** Hypoglossal nerve, Neuromonitoring, Oral tongue cancer surgery, Twelfth cranial nerve, Swallowing

## Abstract

**Background:**

Intraoperative nerve monitoring (IONM) has many applications in different surgical fields. In head and neck surgery, IONM has been used to perform surgery of the parotid, thyroid and parathyroid glands, preserving the facial and recurrent nerves. However, hypoglossal nerve neuromonitoring has not been addressed with such relevance.

**Material and methods:**

A retrospective review of surgeries performed on patients with special tongue and floor of mouth conditions was undertaken to examine the indications that prompted its use. Particular attention was given to the pathology, intraoperative findings and the final outcome of each patient.

**Results:**

Four patients, aged between 6 years and 68 years, with complex oral tongue and floor of mouth lesions were reviewed. Three patients were male, aged 22 years and younger, and two of these patients had oral tongue cancers with previous surgery. Oral tongue and neck conditions are challenging since the functions of the hypoglossal nerve are put at risk. The use of IONM technology allowed us to preserve nerve functions, speech and swallowing.

**Conclusions:**

Although IONM of the hypoglossal nerve is not a common indication in tongue and floor of mouth lesions, under special conditions its application can be extrapolated to challenging surgical cases, like the ones described.

## Background

A number of reports have documented that monitoring a specific nerve during an operation can improve patient outcomes [1-10]. Indeed, intraoperative nerve monitoring (IONM) has been successfully used in surgeries of the skull base and acoustic neuromas [1-4]. In head and neck surgery, IONM has been used in surgeries of the parotid, thyroid and parathyroid glands [5-7]. However, to the best of our knowledge, there are no publications describing IONM in complex lesions of the tongue and floor of mouth.

The hypoglossal nerve is the last nerve to arise from the paired hypoglossal nuclei in the caudal medulla. It exits the cranium through the hypoglossal foramen, travels next to the internal carotid artery and vagus nerve, descends toward the carotid bulb and internal jugular vein, and lies next to the posterior belly of the digastric muscle, beneath the submandibular gland to innervate the extrinsic (genioglossus, hyoglossus and styloglossus) and intrinsic muscles of the tongue. Therefore, lesions to the nerve not only affect the initial process of swallowing, but also speech, coordinated chewing and breathing [11,12]. IONM of the hypoglossal nerve has been reported on proximal lesions located near its exits from the cranium into the neck, such as lesions comprising the cerebellopontine angle or lesions at the skull base [8-10].

In the present article, we describe our experience of four patients treated at the Hospital Pablo Tobón Uribe, Medellin, Colombia, on whom IONM of the hypoglossal nerve was used. We were able to achieve extensive resections with good physiological outcomes and no loss of function with a non-neurosurgical indication.

## Case presentation

A detailed description of Intraoperative neuromonitoring (IONM) of the Hypoglossal nerve is depicted throughout a narrative explanation of four illustrative cases on whom the technique was used . Information about the different steps involved in this novel application of Neuromonitoring is given in order to illustrate the reproducibility of the technology when a surgeon is faced with complex lesions related to the function of the Twelfth cranial nerve.

## Methods

A retrospective review of four patients treated at the Hospital Pablo Tobón Uribe, Medellin, Colombia was undertaken (Table 1). The study was conducted between January 2009 and December 2012. The research protocol was approved by the local Institutional Review Board. Patients were intraoperatively monitored using a NIM™ nerve monitoring system (Medtronic, Jacksonville, FL, USA). To the best of our knowledge, at that time this was the only neuromonitoring system available in Colombia and Latin America. Model NIM™ 2.0 was used in one patient and NIM™ 3.0 in the other three patients. The pathology, intraoperative findings and the final functional outcomes (tongue mobility, speech, swallowing, breathing disorders and duration of tracheostomy) were documented.

**Table 1:** Description of patients with hypoglossal nerve IONM

**Gender, age**	**Diagnosis**	**Surgery**	**Final outcome**
Male, 6 years	Enlarged neck. Hemangiolymphangioma of the right side of neck, floor of mouth and tongue	BND and floor of mouth resection. Previously injured right cranial nerve XII. Second intent to resection	Resection incomplete, since the right nerve was previously injured in a first attempt to resect the tumor. Left cranial nerve XII was left intact with ipsilateral tongue mobility
Male, 8 years	SCC of the anterior oral tongue	Tracheostomy, BND, anterior glossectomy and floor of mouth resection. Reconstruction RFFF	Decannulated 1 month after surgery. Posterior tongue mobility, and able to swallow, speak and articulate
Male, 22 years	Recurrent SCC of the left tongue. Underwent right hemiglossectomy of the right tongue 5 years prior	Tracheostomy, BND, left hemiglossectomy and floor of mouth resection. Reconstruction RFFF	Decannulated 2 weeks after surgery. Remaining oral tongue mobility, slight movement of the RFFF, and able to swallow, speak and articulate
Female, 68 years	Obstructing macroglossia resulting from amyloidosis, secondary to multiple myeloma. Sleep apnea. Not able to swallow solid foods	Tracheostomy, BND, and anterior and posterior midline extended glossectomy	Tongue mobility and able to swallow. Improved sleep apnea. Patient died owing to complications treating the multiple myeloma

*BND* Bilateral neck dissection, *IONM* Intraoperative nerve monitoring, *RFFF* Radial forearm free flap, *SCC* Squamous cell carcinoma.

### Description of the technique

To monitor the twelfth cranial nerve (XII), ground electrodes were placed on the chest or shoulder, following the procedures of other head and neck surgeries where IONM is used. Two sensing electrodes were placed on each side of the tongue either at the beginning of the surgery or once the hypoglossal nerve was exposed in the neck. The nerve was then stimulated with a manual stimulator provided by the manufacturer. A twitch of the tongue on the ipsilateral side, a dull sound and a graphic spike confirmed the integrity of the nerve. Nasotracheal intubation or a tracheostomy was undertaken; tracheostomy was preferred to allow the surgeon space to perform the procedure without having the orotracheal tube obstruct the view. Intubation was achieved with the use of short-acting, rapid-onset muscle relaxant.

## Results

Four patients with complex tongue and/or floor of mouth lesions were monitored intraoperatively with IONM. Table 1 details the diagnosis and final outcome of each patient. The age of patients ranged from 6 years to 68 years. Three patients were male and two of these patients had tongue squamous-cell carcinoma (SCC). There was only one female, the 68-year-old patient.

The first patient was a 6-year-old boy with a hemangiolymphangioma of the right side of the neck, floor of mouth and tongue, and also on the left side, albeit to a lesser degree. A previous attempt to resect the tumor without considering any other alternative was attempted by another surgical team, but was not successful. Since the symptoms worsened, we performed bilateral nerve dissection (BND) with IONM. The larger, right part of the tumor was approached without being able to obtain a signal/response once the nerve was identified; it was later found that the nerve had been injured in the first surgery. The lesion of the neck and floor of mouth was removed. On approaching the left side of the neck, IONM produced a signal once the left hypoglossal nerve was identified and a careful dissection was performed to avoid injuring this working nerve, leaving a small amount of tumor attached to the most anterior part of the nerve.

The second patient was an 8-year-old boy with a lesion on the floor of the mouth and anterior aspect of the tongue. The patient had a history of pulmonary tuberculosis (TB) and was initially given a clinical diagnosis of oral TB. After multiple negative acid-fast bacillus (AFB) smears and cultures, the diagnosis of SCC was established and the patient was referred for surgery. The anterior aspect of the tongue was fixed, suggesting fibrosis or tumor invasion. Therefore, bilateral supraomohyoid neck dissection was performed with IONM.

**Figure 1:**
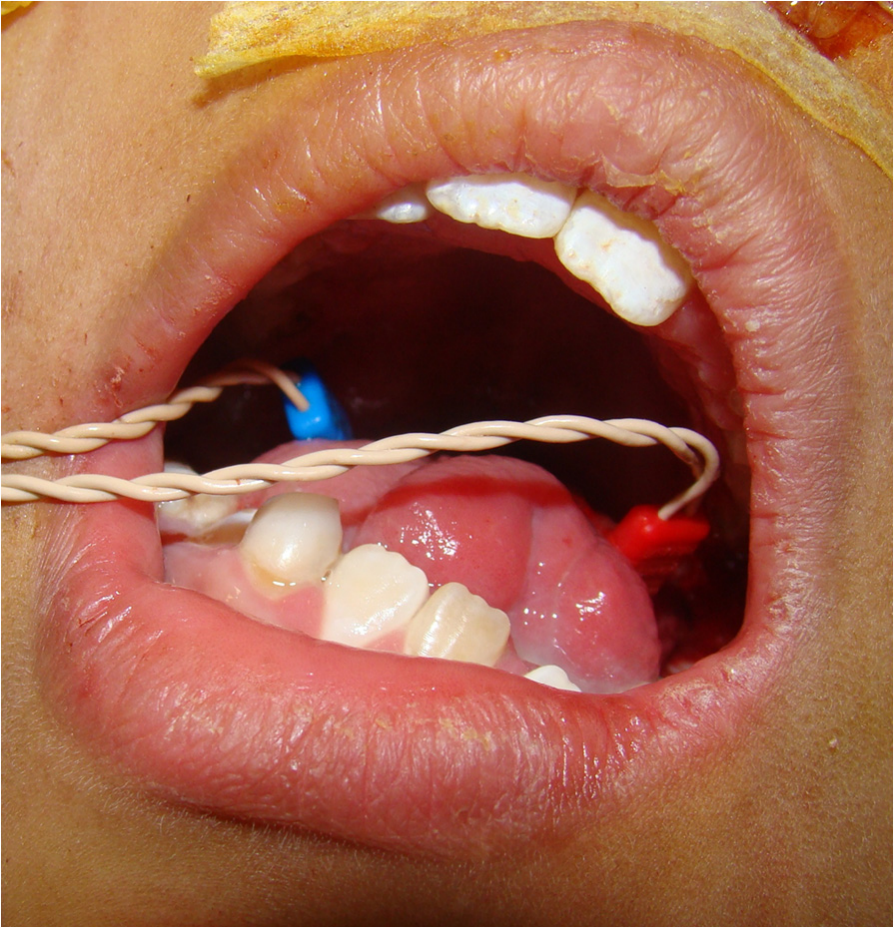
Right and left electrodes are placed on the patient’s tongue, once both hypoglossal trunks were exposed in the neck prior to oral tongue resection.

The electrodes were placed on the most posterior aspect of the oral tongue (Figure 1). Both the main hypoglossal trunks were identified and found to be functionally competent, and an anterior glossectomy with resection of the floor of mouth was performed. To tailor the resection, multiple frozen sections were examined and those containing SCC were excised *en bloc*, regardless of the proximity of a hypoglossal branch; whereas cancer-free areas were preserved. Upon removal of the tumor, stimulation of each hypoglossal nerve branch lead to ipsilateral electromyography (EMG) activity and corresponding contraction of the remaining tongue. The patient was decannulated 1 month after surgery. He was able to swallow a semi-solid diet and was also able to speak. The patient underwent postoperative radiation therapy and was disease-free 14 months after surgery.

The third patient was a 22-year-old man with a history of SCC of the right tongue. The patient had undergone a right hemiglossectomy without complementary radiation therapy 9 years earlier. He presented with a recurrence in his remaining tongue manifesting as a 3 × 3 cm infiltrative lesion on the left tongue, which was found to be SCC on biopsy. We performed a left and right (revision) supraomohyoid neck dissection, and a left hemiglossectomy. During the operation, the electrodes were placed on the remaining right base of the tongue and the most posterior aspect of the left oral tongue. Remarkably, the right hypoglossal nerve responded to the stimulation by moving the base of the tongue to the ipsilateral side. The left nerve was intact and therefore a left hemiglossectomy was performed, and a radial forearm free flap (RFFF) was used to reconstruct the excised tongue. Surgical margins were cancer-free, and there were no intraoperative or postoperative complications. The patient was decannulated 12 days after surgery, and was able to eat, speak and move the flap with significant improvement in phonation compared to preoperative conditions. He completed his radiation therapy and was disease-free 9 months after surgery.

The fourth patient was a 68-year-old woman. The patient was unable to swallow and had severe sleep apnea with failed nocturnal continuous positive airway pressure (CPAP). She was operated on owing to a large obstructing macroglossia resulting from amyloidosis. A partial glossectomy under IONM was performed by placing the electrodes on the most posterior part of the patient’s oral tongue. Forty percent of the patient’s oral tongue together with the most anterior aspect of the base of tongue was excised in a rhomboid fashion. Pathological examination of the removed tissues confirmed amyloidosis but also showed multiple myeloma. Following surgery, the patient remained unable to swallow and was was not able to be decannulated as her base of tongue remained swollen. In preparation for her first chemotherapy cycle, a surgical gastrostomy with placement of a G-tube was performed (the endoscope could not be advanced to the esophagus). Unfortunately the patient died owing to complications treating the multiple myeloma.

## Discussion

IONM is a technique that allows surgeons to preserve critical nerves during surgical procedures. Critical anatomical areas can be securely preserved by testing a particular structure before operating, which can avoid complications and provide the surgical team with the final status of the nerve [5,7,9,13].

IONM indications are varied since its early use in acoustic neuroma has spread to other surgical specialties, such as neurosurgery, skull base, and head and neck surgery [1-4]. High volume tumors and recurrent lesions present a challenge to surgeons and patients, who anticipate the best results regardless of previous conditions. Head and neck surgeons use IONM in parotid surgery to monitor the facial nerves, especially in thyroid and parathyroid surgery of the recurrent and superior laryngeal nerves [5-7]. We extrapolated our experience of IONM in head and neck surgery with four difficult cases of uncommon tongue and floor of mouth lesions. Three out of four of these patients presented with previous surgery.

Regarding the hypoglossal nerve, its anatomical relations and involvement by lesions in the neck, an experienced head and neck surgeon should be able to identify the main trunk in the neck; Walshe stated that ‘there is no substitution for a good anatomical knowledge when identifying the hypoglossal nerve in the neck’. The hypoglossal nerve is identified in the neck owing to its size and usual anatomical location, although anatomical variations of the nerve have been described [11,13].

Hypoglossal nerve monitoring can be achieved by head and neck surgeons familiar with neuromonitoring of the facial and recurrent nerves, by following the procedures of other head and neck surgeries where IONM is used [5]. This is not a common indication of IONM in head and neck surgery, and most published articles on IONM are related to neurosurgery [1-3].

Different from various neurosurgical indications, it could be questioned whether head and neck surgeons should be required to schedule surgery with IONM for every patient with a tongue lesion. In thyroid surgery, there is a debate regarding the need to use the technology for every case regardless of different conditions (tumor volume, paralyzed nerve, and so on) [5-7]. In patients undergoing a classic hemiglossectomy for the first time for a tongue or floor of mouth cancer, the use of IONM should not be used. It is clear that the technology would not change the procedure and would not provide additional information necessary for the surgery. Similarly to thyroid surgery using IONM to monitor the hypoglossal nerve, IONM increases the cost of the procedure compared to when the technology is not used; an important factor to consider in today’s changing politics of health care [14,15]. However, under special conditions and quite rare cases like the ones described with large lesions, and especially with prior surgery, complicated anatomical conditions, and scar and fibrotic tissue, surgeons should consider this available technology and its indications, balance the advantages and disadvantages, and reach an informed decision on a case-by-case basis.

As described, two of the young male patients had SCC and, in these difficult cases of oral tongue and floor of mouth lesions, the surgical team did not want to compromise positive margins in order to preserve function. For the avoidance of doubt, we do not use the IONM technology on regular cases. Prior to sectioning the tissue, multiple frozen sections were taken from the normal-appearing tongue or floor of mouth mucosa tissue, to identify the minor hypoglossal nerve branches and direct the resections of the lesions. If they were identified near the tumor they were sacrificed, regardless of proximity to a nerve branch. It was rewarding to know that at the end of each case, the young patients had good amplitude wave EMG responses in both nerves and a positive functional outcome of mobility of the tongue itself. These positive results allowed us to remove the tracheostomy in two out of three patients; only the fourth patient remained with the tracheostomy cannula in place, even though the patient’s tongue remained mobile with functional hypoglossal nerve.

We choose to place the electrodes directly onto the posterior aspect of the anterior tongue instead of the extrinsic muscles in order to maintain the intrinsic musculature and obtain an optimum result. Energy to stimulate the nerves varied from 0.5 mA to 1.0 mA, with an average of 0.8 mA [8-10].

To the best of our knowledge, Walshe’s study on hypoglossal nerve stimulation is the only published article with true head and neck indications [8], however, the article describes patients with submandibular gland resections and neck dissections. Similarly to Walshe’s study, our retrospective review of four patients is small and, therefore, the relevance is limited by the few cases reported. A prospective study recruiting more patients with lesions like the ones described will be difficult, since our regular patients requiring a hemiglossectomy will not be scheduled with this type of technology.

Finally, the system helped us to identify a previously injured nerve on a young patient with a large volume lesion on the oral tongue and floor of mouth. This was not detected prior to surgery since the patient was barely moving his tongue displaced by the hemangiolymphangioma. The surgical team was able to remove the lesion and preserve the left working hypoglossal nerve.

## Conclusions

Head and neck surgery applications of IONM have been mainly restricted to thyroid, parathyroid and parotid surgery. Although monitoring the hypoglossal nerve is not a regular indication of this technique, surgeons should be aware of the potential ‘nonconventional’ applications that the technology can offer to challenging surgical cases like the ones described.

## Consent

Written informed consent was obtained from the patients for publication of this case report and any accompanying images. A copy of the written consent is available for review by the Editor-in-Chief of this journal.

## Abbreviations

AFB: Acid-fast bacillus; BND: Bilateral nerve dissection; CPAP: Continuous positive airway pressure; EMG: Electromyography; IONM: Intraoperative nerve monitoring; RFFF: Radial forearm free flap; SCC: Squamous-cell carcinoma; TB: Tuberculosis.

## Competing interests

CSD and JPD gave instructional courses on neuromonitoring to surgeons in Colombia and Latin American in 2012, sponsored by Medtronic. However, this study was not supported by any company.

## Authors’ contributions

CSD, AL, AP performed the operations. DU assisted the surgeries. JPD gave input in neuromonitoring. All authors read and approved the final manuscript.
